# 3-Aminothiophene-2-Acylhydrazones: Non-Toxic, Analgesic and Anti-Inflammatory Lead-Candidates

**DOI:** 10.3390/molecules19068456

**Published:** 2014-06-20

**Authors:** Yolanda Karla Cupertino da Silva, Christian Tadeo Moreno Reyes, Gildardo Rivera, Marina Amaral Alves, Eliezer J. Barreiro, Magna Suzana Alexandre Moreira, Lídia Moreira Lima

**Affiliations:** 1 LaFI—Laboratório de Farmacologia e Imunidade, Instituto de Ciências Biológicas e da Saúde, Universidade Federal de Alagoas, Maceió 57072-900, AL, Brazil; E-Mail: yolandakarla@yahoo.com.br; 2 Laboratório de Avaliação e Síntese de Substâncias Bioativas—LASSBio, Programa de Pesquisa em Desenvolvimento de Fármacos, Instituto de Ciências Biomédicas, Universidade Federal do Rio de Janeiro, PO Box 68024, Rio de Janeiro 21944-902, RJ, Brazil; E-Mails: cristian@buscarsalud.com (C.T.M.R.); marinamaral@hotmail.com (M.A.A.); ejbarreiro@ccsdecania.ufrj.br (E.J.B.); 3 Centro de Biotecnologia Genomica, Instituto Politecnico Nacional, Boulevard del Maestro, s/n, 88710 Reynosa, Mexico; E-Mail: gildardors@hotmail.com

**Keywords:** anti-inflammatory, toxicity, acylhydrazone, analgesic, arthritis, privileged structure

## Abstract

Different chemotypes are described as anti-inflammatory. Among them the *N*-acylhydrazones (NAH) are highlighted by their privileged structure nature, being present in several anti-inflammatory drug-candidates. In this paper a series of functionalized 3-aminothiophene-2-acylhydrazone derivatives **5a**–**i** were designed, synthesized and bioassayed. These new derivatives showed great anti-inflammatory and analgesic potency and efficacy. Compounds **5a** and **5d** stand out in this respect, and were also active in CFA-induced arthritis in rats. After daily treatment for seven days with **5a** and **5d** (50 µmol/Kg), by oral administration, these compounds were not renal or hepatotoxic nor immunosuppressive. Compounds **5a** and **5d** also displayed good drug-scores and low risk toxicity calculated *in silico* using the program OSIRIS Property Explorer.

## 1. Introduction

**Figure 1:**
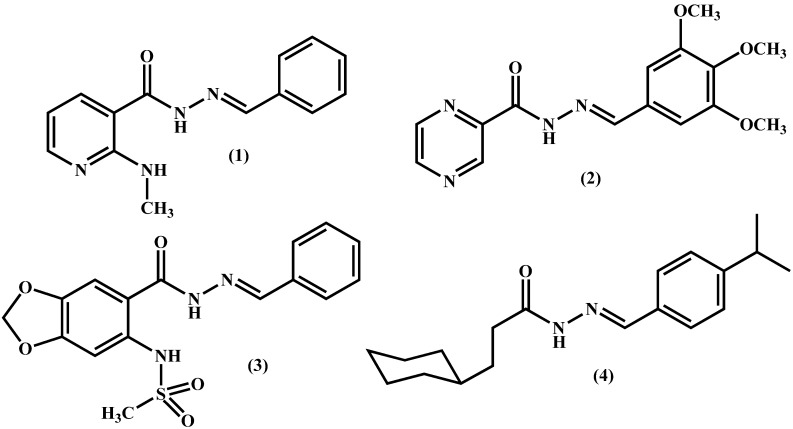
Examples of anti-inflammatory lead-candidates presenting the privileged *N*‑acylhydrazone structure.

Inflammation is an adaptive response that is triggered by noxious stimuli and conditions, such as infection and tissue injury, and can be classified into acute and chronic [1,2]. The role of inflammation in the etiology of several diseases is well-documented [3,4]. Many pathways are involved in acute and chronic inflammatory processes, among them the cyclooxygenase (COX) and p38 mitogen-activated protein kinase (p38 MAPK). These enzymes have been studied as important targets for the design of novel anti-inflammatory drugs [5,6,7,8]. COX and its isoenzymes (COX-1; COX-2 and COX-3) are the molecular targets of non-steroidal anti-inflammatory drugs (e.g., indomethacin, diclofenac and celecoxib). COX-3 is believed to be responsible for the antipyretic and analgesic effects of dipyrone and paracetamol [9]. Distinct chemotypes are described as anti-inflammatory, and among them the *N*-acylhydrazones (NAH) are especially noteworthy [10]. These privileged structures [11], exemplified by compounds **1**–**4** (Figure 1), are present in several anti-inflammatory drug-candidates acting by different mechanism of action [12,13,14,15,16,17,18]. 

**Figure 2:**
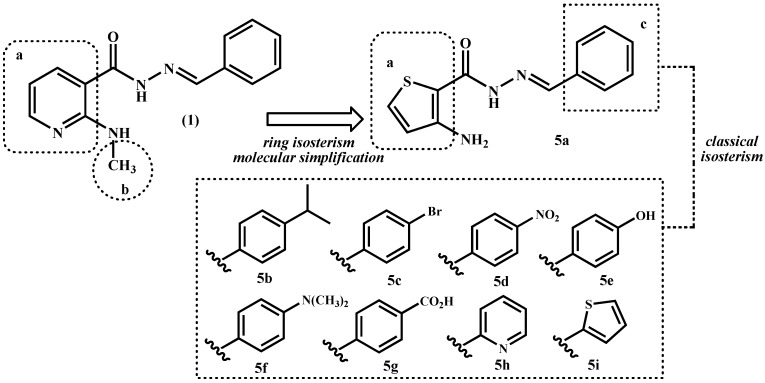
Design concept of the 3-aminothiophene-2-acylhydrazone derivatives **5a**–**i**.

In a continuous effort to identify new anti-inflammatory drug-candidates a series of functionalized 3-aminothiophene-2-acylhydrazone derivatives **5a**–**i** were designed by molecular modifications of the prototype **1**. These modifications aimed to preserve the *N*-acylhydrazone moiety (CONHN=CH) and were based on application of ring isosterism between the pyridine and thiophene nucleus, and on molecular simplification with consequent elimination of the methyl group of the original prototype **1** (Figure 2). The aromatic pattern of compound **5a** was later modified following classical isosteric replacement of monovalent groups and equivalent rings exchange [19] (Figure 2).

## 2. Results and Discussion

### 2.1. Chemistry

**Scheme 1:**
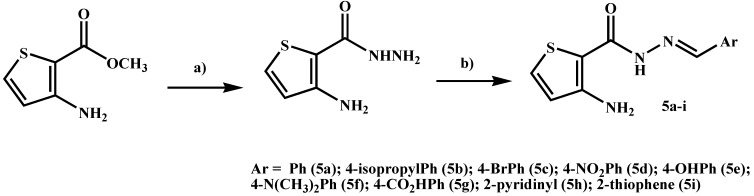
Synthesis of 3-aminothiophene-2-acylhydrazones derivatives **5a**–**i**.

The functionalized 3-aminothiophene-2-acylhydrazone derivatives **5a**–**i** were synthesized as depicted in Scheme 1, following previously described methodology [20,21,22,23]. Compounds were characterized by IR, ^1^H- and ^13^C-NMR and mass spectroscopy. The purity was determined by HPLC. The stereochemistry of the imine double bond (N=CH) was assigned as the *E*-isomer. This isomer was assigned based on the chemical shift of the imine-hydrogen singlet signal visualized in the ^1^H-NMR spectra of NAH derivatives **5a**–**i** and considering similar data previously reported in the literature [21,22,23].

*Reagents and Conditions*: (**a**) N_2_H_4_.H_2_O (80%), EtOH, reflux, 24 h, 64%; (**b**) ArCHO, EtOH, HCl (cat), r.t., 1 h, 47%–95%.

### 2.2. In Silico Drug-Like Profile and Toxicity

**Figure 3:**
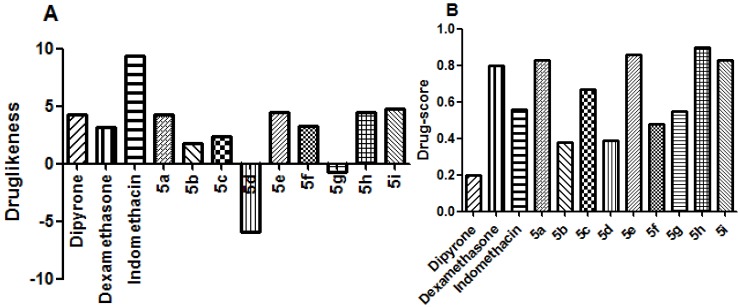
Drug-like profile of 3-aminothiophene-2-carbohydrazide derivatives **5a**–**i** calculated by Osiris Program. (**A**) druglikeness; (**B**) drug-score values.

In order to investigate the potential toxicity of *N*-acylhydrazones **5a**–**i** and their drug-like profile, these compounds were evaluated *in silico* using the Program OSIRIS Property Explorer [24]. As showed in Figure 3A,B, with exception of compounds **5g** and **5d**, the 3-aminothiophene-2-acylhydrazones **5a**–**i** were predicted to have similar druglikeness as dipyrone and dexamethasone, and inferior to that of indomethacin. Examining the drug-score parameter, OSIRIS predicted all compound **5a**–**i** as being better than dipyrone. Likewise, derivatives **5a**, **5e**, **5h** and **5i** were predicted to have similar drug-score as dexamethasone, while compounds **5c**, **5f** and **5g** were similar to indomethacin. Despite the close structural similarity between compounds **5a**–**i**, the *in silico* study predicted different toxic profiles (Table 1). Compounds **5a**, **5c**, **5d**, **5g** and **5i** were predicted with low risk of toxicity; whereas derivatives **5b**, **5e**, **5f** and **5h** showed moderate to high theoretical toxicity risk, for at least one of the parameters analyzed by the Osiris program.

**Table 1:** *In silico* toxicity of 3-aminothiophene-2-carbohydrazide derivatives **5a**–**i** calculated using Osiris program [24,25].

Compound	Theoretical Toxicity Risks *			
	Mutagenic	Tumorigenic	Irritant	Reproductive Effects
**Dipyrone**	3	3	1	3
**Dexamethasone**	1	1	1	1
**Indomethacin**	1	1	1	1
**5a**	1	1	1	1
**5b**	1	1	3	1
**5c**	1	1	1	1
**5d**	1	1	1	1
**5e**	1	1	2	2
**5f**	1	3	1	1
**5g**	1	1	1	1
**5h**	3	1	1	1
**5i**	1	1	1	1

* Theoretical toxicity risks calculated using Osiris program. The scale of side effects is low (1), medium (2), and high (3) toxicity profile.

### 2.3. Biological Assays

To investigate the antinociceptive profile of compounds **5a**–**i**, the dose-response of these derivatives in the acetic acid-induced abdominal constriction test was determined [26]. Mice were treated with compounds **5a**–**i** in doses of 100, 30, 10, 3 and 1 µmol/Kg, by oral administration, using dipyrone as standard drug. As exemplified in Table 2, with the exception of compounds **5e** and **5g**, that were inactive, the *N*-acylhydrazone derivatives **5a**–**i** showed high potency and efficacy. Compounds **5a** (ID_50_ = 3.5 ± 0.1 µmol/Kg), **5c** (ID_50_ = 2.3 ± 0.4 µmol/Kg), **5d** (ID_50_ = 2.6 ± 0.5 µmol/Kg) and **5h** (ID_50_ = 2.5 ± 0.4 µmol/Kg) were more potent than the standard dipyrone (ID_50_ = 11.4 ± 4.9 µmol/Kg).

**Table 2:** ID_50_s of compounds **5a**–**i** (100, 30, 10, 3 and 1 µmol/Kg, p.o.) and dipyrone (100, 30, 10, 3 and 1 µmol/Kg, p.o.) in the 0.6% acetic acid-induced abdominal constrictions in mice assay, for a period of 25 min.

Compound	ID_50_ (µmol/Kg ± S.E.M.)	Emax (% ± S.E.M.)
**Dipyrone**	11.4 ± 4.9	75.2 ± 5.8
**5a**	3.5 ± 0.1	72.6 ± 4.1
**5b**	6.1 ± 2.2	64.3 ± 8.5
**5c**	2.3 ± 0.4	76.7 ± 5.2
**5d**	2.6 ± 0.5	81.0 ± 5.7
**5e**	>100	-
**5f**	32.8 ± 17.3	74.5 ± 9.6
**5g**	>100	-
**5h**	2.5 ± 0.4	84.1 ± 5.6
**5i**	7.8 ± 3.2	86.8 ± 5.2

**Figure 4:**
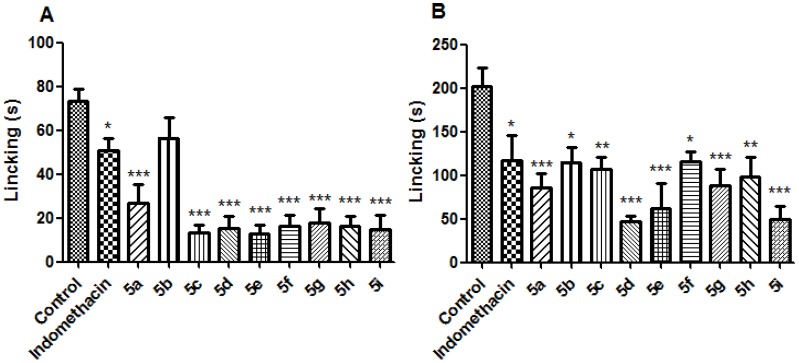
Effect of compounds **5a**–**i** (30 µmol/Kg, p.o.) and indomethacin (10 µmol/Kg, p.o) on formalin (2.5%) test in mice. (**A**) neurogenic phase; (**B**) inflammatory phase. Statistical differences between the treated and the control groups were evaluated by ANOVA and Dunnett tests and the asterisks denote the levels of significance in comparison with control groups. *** *p* < 0.001; ** *p* < 0.01 and * *p* < 0.05.

Next, these compounds were evaluated in a formalin test [27] using a screening dose of 30 µmol/Kg (*per os*). All compounds were able to reduce the nociception response in phase 1 (neurogenic) and phase 2 (inflammatory) of formalin test. Compounds **5d** and **5i** produced noteworthy inhibition during the inflammatory phase of the formalin assay (Figure 4A,B).

The anti-inflammatory activity of compounds **5a**–**i** was evaluated using a carrageenan-induced peritonitis test [28]. Animals were treated with **5a**–**i** by oral administration in doses of 100, 30, 10, 3 and 1 µmol/Kg. The anti-inflammatory potency of compounds **5a**–**i** is listed in Table 3. All compounds, with exception of **5e** and **5i** promoted a significant reduction of cell recruitment, being derivatives **5a** (ID_50_ = 7.2 ± 1.8 µmol/Kg) and **5d** (ID_50_ = 5.2 ± 2.0 µmol/Kg) the most promising, showing anti-inflammatory potency and efficacy similar to the standard indomethacin (Table 3).

**Figure 5:**
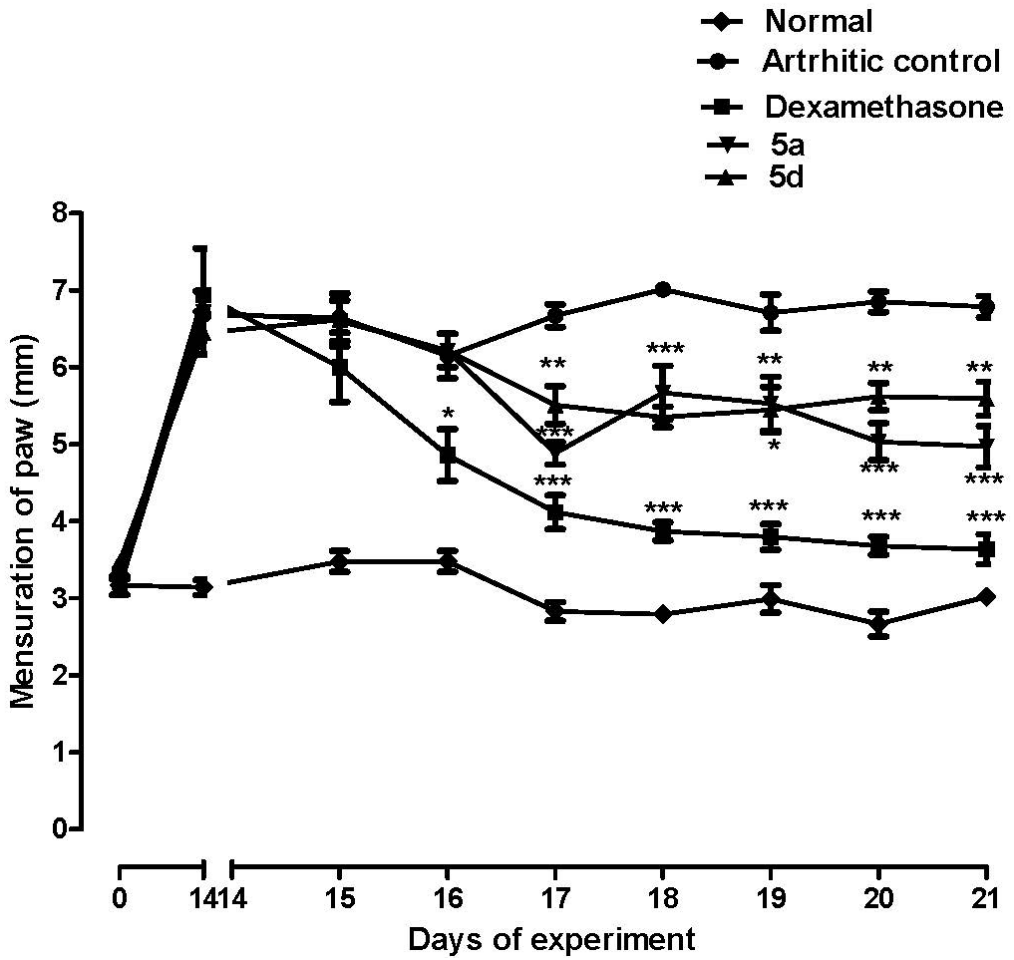
Effect of compounds **5a** (50 µmol/Kg, p.o.) and **5d** (50 µmol/Kg, p.o.) and dexamethasone (5 µmol/Kg; p.o.) on the CFA-induced arthritis in rats. Each point represents the mean ± S.E.M. of six animals. Statistical differences between the treated and the control groups were evaluated by ANOVA and Dunnett tests and the asterisks denote the levels of significance in comparison with control groups. *** *p* < 0.001; ** *p* < 0.01 and * *p* < 0.05.

One the anti-inflammatory profile of 3-aminothiophene-2-acylhydrazones **5a**–**i** was confirmed in a murine acute inflammatory model, compounds **5a** and **5d** were selected in order to investigate their activity in a chronic model of inflammation. Therefore, these compounds (50 µmol/Kg, pathway orally) were evaluated in an arthritis model induced by complete Freund’s adjuvant (CFA) in rats [29], and the results were compared to those obtained for the standard dexamethasone (5 µmol/Kg, pathway orally, Figure 5). As demonstrated in Figure 5, the daily treatment for seven days with compounds **5a** and **5d** was able to reduce paw edema on the 17th to 21st days of experiment, although, this reduction was less prominent than that caused by the standard dexamethasone.

**Table 3:** ID_50_ of compounds **5a**–**i** (100, 30, 10, 3 and 1 µmol/Kg, p.o.) and indomethacin (100, 30, 10, 3 and 1 µmol/Kg, p.o.) on the carrageenan-induced peritonitis in mice.

Compound	ID_50_ (µmol/Kg ± S.E.M.)	Emax (% ± S.E.M.)
**Indomethacin**	3.3 ± 1.0	73.8 ± 5.1
**5a**	7.2 ± 1.8	69.7 ± 12.1
**5b**	15.9 ± 6.3	73.9 ± 11.7
**5c**	8.5 ± 4.2	72.7 ± 5.9
**5d**	5.2 ± 2.0	70.3 ± 5.2
**5e**	>100	-
**5f**	8.8 ± 6.1	62.4 ± 5.8
**5g**	15.3 ± 12.5	75.1 ± 1.6
**5h**	20.2 ± 17.2	76.4 ± 10.1
**5i**	>100	-

**Figure 6:**
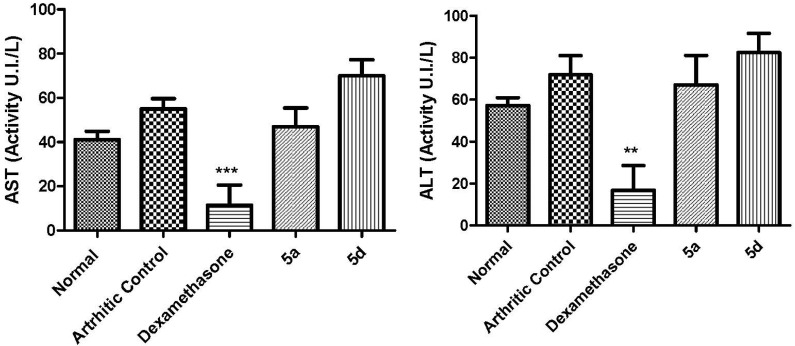
Effect of compounds **5a** (50 µmol/Kg, p.o) and **5d** (50 µmol/Kg, p.o) and dexamethasone (5 µmol/Kg) on serum ALT and AST levels.Statistical differences between the treated and the control groups were evaluated by ANOVA and Dunnett tests and the asterisks denote the levels of significance in comparison with control groups. *** *p* < 0.001 and ** *p* < 0.01.

**Figure 7:**
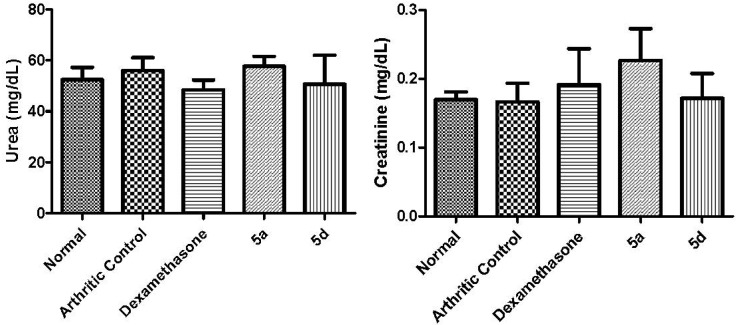
Effect of compounds **5a** (50 µmol/Kg, p.o.) and **5d** (50 µmol/Kg, p.o.) and dexamethasone (5 µmol/Kg, p.o.) on serum Urea and Creatinine levels.Statistical differences between the treated and the control groups were evaluated by ANOVA.

After the 21st day of the arthritis experiment, animals were anesthetized and blood was collected to investigate whether treatment with **5a** and **5d** had generated any liver or kidney toxicity. To assess liver and renal functions the AST/ALT and urea/creatinine levels were determined on serum of animals treated with **5a** and **5d** (Figure 6 and Figure 7). No alterations were observed in the levels of AST/ALT (Figure 6) and/or urea/creatinine (Figure 7). These data indicated that, in these conditions, compounds **5a** and **5d** were neither hepatotoxic nor nephrotoxic. After the daily treatment for seven days with **5a** and **5d** (50 µmol/Kg, pathway orally) the stomach of the animals was macroscopically analyzed [30] and no signal of redness or bleeding was observed (data not shown).

**Figure 8:**
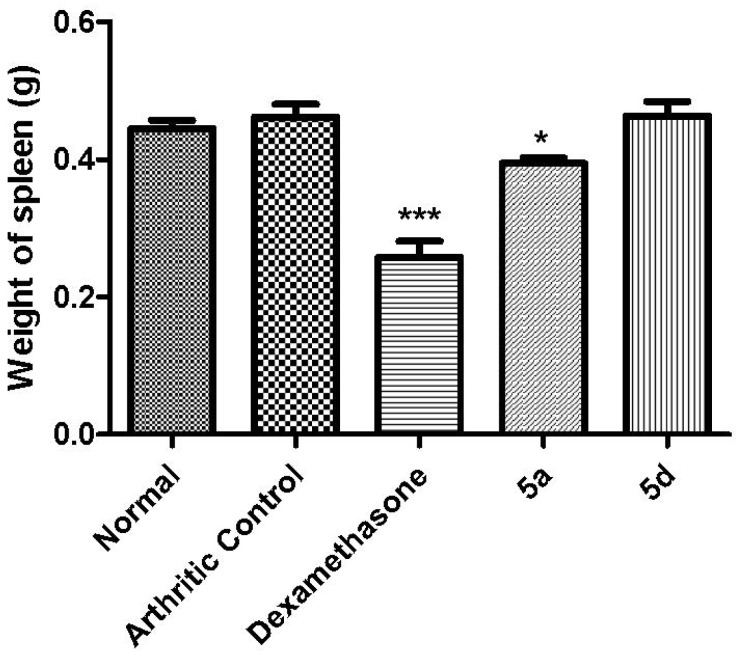
Effect of compounds **5a** (50 µmol/Kg, p.o.) and **5d** (50 µmol/Kg, p.o.) and dexamethasone (5 µmol/Kg, p.o.) on weight of spleen.Statistical differences between the treated and the control groups were evaluated by ANOVA and Dunnett tests and the asterisks denote the levels of significance in comparison with control groups. *** *p* < 0.001 and * *p* < 0.05.

In order to discount an eventual immunosuppressive profile and considering that several indicators of immunosuppression can be observed in standard nonclinical toxicology studies, such as alterations in organ weight and hypocellularity of immune system tissues [31,32,33], spleens of animals treated daily for seven days with compounds **5a**, **5d** and dexamethasone were also analyzed. No change in spleen weight was observed after treatment with **5d** **(**50 µmol/Kg, p.o.), whereas a slight alteration was detected with **5a** **(**50 µmol/Kg, p.o.). These results indicate that **5d** doesn’t promote immunosuppression, while the standard drug dexamethasone does (5 µmol/Kg, p.o.), as indicated by its ability to change the spleen’s weight (Figure 8).

## 3. Experimental

### 3.1. General Information

Acetic acid (Merck, São Paulo, SP, Brazil), arabic gum (Sigma-Aldrich, São Paulo, SP, Brazil), carrageenan (Sigma-Aldrich), complete Freund’s adjuvant (CFA) (Sigma-Aldrich), dipyrone (Sigma-Chemical, São Paulo, SP, Brazil), indomethacin (Merck Sharp & Dohme, Barueri, SP, Brazil) and dexamethasone (Sigma-Chemical) were obtained from commercial sources. A solution of 2.5% formalin was prepared with formaldehyde (Merck) in saline (NaCl 0.9%). (Merck). Kits were used for biochemical dosage: AST, ALT, urea and creatinin (Doles-Brazil, Goiania, GO, Brazil). The caliper was used to measure the paws (Mitutoyo, Santo Amaro, SP, Brazil).

### 3.2. General Procedure for the Preparation of 3-Aminothiophene-2-Carbohydrazide [23]

To a solution of methyl 3-aminothiophene-2-carboxylate (1 mmol) in ethanol (5 mL) was added hydrazine monohydrate (35 mmol). The reaction mixture was maintained under reflux for 24 h, at which time TLC (hexane‒ethyl acetate (1:1)) indicated the end of the reaction. Then the reaction mixture was poured on ice and the resulting white precipitate was filtered out affording the title compound. This compound was prepared by Reinecke and coworkers using a similar procedure [22]. ^1^H-NMR (400 MHz, DMSO-*d*_6_) δ (ppm): δ 4.3 (s, 2H, NH_2_); 6.3 (s, 2H, Ar-NH_2_); 6.5 (d, 1H, H4), 7.4 (d, 1H, H3); 8.6 (s, 1H, NH). ^13^C-NMR (50 MHz, DMSO-*d*_6_) δ (ppm): 165 (CO); 152 (CNH_2_); 128 (C4); 120 (C1); 99 (C3). MS: *m/z* = 158 [M + H]^+^.

### 3.3. General Procedure for the Preparation of 3-Aminothiophene-2-Carbohydrazide Derivatives **5a**–**i**

Under anhydrous conditions 3-aminothiophene-2-carbohydrazide (1.0 mmol), ethanol (5 mL), the corresponding benzaldehyde derivative (1.0 mmol) and a catalytic amount of hydrochloric acid were mixed together and stirred for 1 h at reflux and then the solvent was evaporated under reduced pressure. Next the reaction mixture was added to ice and the precipitate obtained was purified by column chromatography on silica gel with a CH_2_Cl_2_/MeOH 2% mobile phase.

*3-Amino-N'-[(1(E)-phenylmethylene]-2-thiophenecarbohydrazide* (**5a**; LASSBio-1660). This compound was previously prepared by Huddleston and coworkers [22]. Yield: 48%; white solid; m.p. 170–173 °C; IR (KBr) cm^−1^: 3049 (ν NH), 1639 (ν CO); ^1^H-NMR (400 MHz, DMSO-*d*_6_) δ (ppm): δ 4.45 (s, 2H, NH_2_), 6.68 (d, 1H, H5), 7.39 (m, 3H, H2', H3' & H4'), 7.62 (d, 1H, H4'), 7.74 (d, 2H, H1' & H2'), 8.04 (s, 1H, N=CH), 11.30 (s, 1H, NH) ppm; 96% purity by HPLC (R.T. = 4.2 min; CH_3_CN‒H_2_O (70:30)); MS: *m/z* = 246 [M + H]^+^. These data are in agreement with the previous report [27].

*3-Amino-N'-[(1(E)-(4-isopropylphenyl)methylene]-2-thiophenecarbohydrazide* (**5b**, LASSBio-1661). Yield: 97%; white solid; m.p. 155–157 °C; IR (KBr) cm^−1^: 3301 (ν NH), 1638 (ν CO), 1599 (ν CN); ^1^H NMR (400 MHz, DMSO-*d*_6_) δ (ppm): δ 1.34 (s, 3H, CH_3_), 1.36 (s, 3H, CH_3_), 2.98 (m, 1H, H4), 4.55 (s, 2H, NH_2_), 6.62 (d, 1H, H3), 7.38 (d, 2H, H2' & H4'), 7.75 (d, 2H, H1' & H5'), 8.11 (s, 1H, N=CH), 11.3 (s, 1H, NH) ppm; 97% purity in HPLC (R.T. = 7.3 min; CH_3_CN‒H_2_O (70:30)); MS: *m/z* = 288 [M + H]^+^.

*3-Amino-N'*-*[(1*(*E)-(4-bromophenyl)methylene]-2-thiophenecarbohydrazide* (**5c**, LASSBio-1654). Yield: 62%; yellow solid; m.p. 230–233 °C; IR (KBr) cm^−1^: 3290 (ν NH), 1637 (ν CO), 1597 (ν CN); ^1^H-NMR (400 MHz, DMSO-*d*_6_) δ (ppm): 6.63 (d, 2H, H2' & H3'), 7.0 (s, 2H, NH_2_), 7.5 (d, 2H, H1' & H4'), 7.58–7.73 (m, 2 H, 1H, H3 & H4), 8.0 (s, 1H, N=CH), 11.29 (s, 1H, NH); ^13^C-NMR (50 MHz, DMSO-*d_6_*) δ (ppm): 165 (CO), 157 (C4'), 141 (C2), 134 (HC=N), 132 (C3' & C5'), 129 (C2' & C6'), 122 (H4), 119 (H3), 96 (C1'); 99% purity by HPLC (R.T. = 5.9 min; CH_3_CN‒H_2_O (70:30)); MS: *m/z* = 323 [M + H]^+^.

*3-Amino-N'-[(1(E)-(4-nitrophenyl)methylene]-2-thiophenecarbohydrazide* (**5d**, LASSBio-1656). Yield: 95%; orange solid; m.p. 255–257 °C; IR (KBr) cm^−1^: 3369 (ν NH), 1645 (ν CO), 1586 (ν CN); ^1^H-NMR (400 MHz, DMSO-*d*_6_) δ (ppm): δ 4.62 (s, 2H, NH_2_), 6.7 (d, 1H, H5), 7.7 (d, 1H, H4), 8.0 (2, 2H, H3' & H5'), 8.1 (s, 1H, N=CH), 8.3 (m, 3H, H4, H2' & H6'), 11.6 (d, 1H, NH); 98% purity by HPLC (R.T. = 4.1 min; CH_3_CN‒H_2_O (70:30)); MS: *m/z* = 291 [M + H]^+^.

*3-Amino-N'-[(1(E)-(4-hidroxyphenyl)methylene]-2-thiophenecarbohydrazide* (**5e**, LASSBio-1657). Yield: 84%; yellow solid; m.p. 245–248 °C; IR (KBr) cm^−1^: 3289 (ν NH), 1637 (ν CO), 1581 (ν NH); ^1^H-NMR (400 MHz, DMSO-*d*_6_) δ (ppm): δ 3.87 (s, 2H, NH_2_), 6.62 (s, 1H, H5), 6.84 (d, 2H, C3' & C5'), 7.56-7.71 (m, 3H, H4, H2' & H6'), 7.92 (s, 1H, N=CH), 9.89 (s, 1H, OH), 11.02 (s, 1H, NH); ^13^C-NMR (50 MHz, DMSO-*d_6_*) δ (ppm): 165 (CO), 159 (C3), 157 (C4'), 142 (HC=N), 134 (C5), 129 (C3' & C5'), 126 (C4), 119 (C1'), 116 (C2' & C6'); 99% purity by HPLC (R.T. = 3.0 min; CH_3_CN‒H_2_O (70:30)); MS: *m/z* = 262 [M + H]^+^.

*3-Amino-N'-[(1(E)-(4-dimethylaminophenyl)methylene]-2-thiophenecarbohydrazide* (**5f**, LASSBio-1653). Yield: 73%; brown solid; m.p. 180–184 °C; IR (KBr) cm^−1^: 3301 (ν NH), 1629(ν CO), 1597 (ν NH); ^1^H-NMR (400 MHz, DMSO-*d*_6_) δ (ppm): δ 2.95 (s, 6H, N(CH_3_)_2_), 3.43 (s, 2H, NH_2_), 6.62 (d, 1H, H5), 6.76 (d, 2H, H3' & H5'), 7.55–7.60 (m, 3H, H4, H2' & H6'), 7.89 (s, 1H, N=CH), 10.95 (s, 1H, NH) ppm; ^13^C-NMR (50 MHz, DMSO-*d*_6_) δ (ppm): 165 (CO), 157 (C4'), 151 (C3), 143 (HC=N), 134 (C5), 128 (C3' & C5'), 122 (C2), 119 (C4), 112 (C2' & C6'), 97 (C1); 93% purity by HPLC (R.T. = 4.5 min; CH_3_CN‒H_2_O (1:1)); MS: *m/z* = 289 [M + H]^+^.

*3-Amino-N'-[(1(E)-(4-carboxyphenyl)methylene]-2-thiophenecarbohydrazide* (**5g**, LASSBio-1655). Yield: 77%; yellow solid; m.p. >300 °C; IR (KBr) cm^−1^: 3347 (ν NH), 1692 (ν CO), 1589 (ν CO); ^1^H-NMR (400 MHz, DMSO-*d*_6_) δ (ppm): 3.7 (s, 2H, NH_2_), 6.62 (d, 1H, H5), 7.59 (d, 1H, H4), 7.84 (d, 2H, C3' & C5'), 7.99 (d, 2H, C2' & C6'), 8.07 (s, 1H, N=CH), 11.45 (s, 1H, NH) ppm; 99% purity by HPLC (R.T. = 2.8 min; CH_3_CNH_2_O (1:1)). MS: *m/z* = 290 [M + H]^+^.

*3-Amino-N'-[(1(E)-(2-pyridinyl)methylene]-2-thiophenecarbohydrazide* (**5h**, LASSBio-1659). Yield: 53%; yellow solid; m.p. 226–228 °C; IR (KBr) cm^−1^: 3288 (ν NH), 1633 (ν CO), 1579 (ν NH); ^1^H-NMR (400 MHz, DMSO-*d*_6_) δ (ppm): 3.5 (s, 2H, NH_2_), 6.64 (d, 1H, C5), 7.36 (t, 1H, H4'), 7.59 (d, 1H, H4), 7.89 (d, 1H, H6'), 8.06 (m, 2H, H3' & H5'), 8.5 (s, 1H, N=CH), 11.42 (s, 1H, NH) ppm; 98% purity by HPLC (R.T. = 3.1 min; CH_3_CN‒H_2_O (1:1)); MS: *m/z* = 247 [M + H]^+^.

*3-Amino-N'-[(1(E)-(2-thienyl)methylene]-2-thiophenecarbohydrazide* (**5i**, LASSBio-1658). Yield: 79%; yellow solid; m.p. 221–225 °C; ^1^H-NMR (400 MHz, DMSO-*d*_6_) δ (ppm): 6.60 (s, 1H, H4'), 4.65 (s, 2H, NH_2_), 7.09 (s, 1H, H5'), 7.37 (s, 1H, H3'), 7.55–7.60 (m, 2H, H4 & H5), 8.20 (s, 1H, N=CH), 11.16 (s, 1H, NH); 99% purity by HPLC (R.T. = 3.8 min; CH_3_CN‒H_2_O (1:1)); MS: *m/z* = 252 [M + H]^+^.

### 3.4. In Silico Toxicological Evaluation and Drug-Like Profile

The *in silico* toxicity and drug-like profile of 3-aminothiophene-2-acylhydrazone derivatives **5a**–**i** were calculated using the Program OSIRIS Property Explorer [24]. Data were generated on-line in the Osiris Program, accessed by the link (http://www.organic-chemistry.org/prog/peo/) [25] and represented by toxicity risks (mutagenic, irritant, tumorigenic and reproductive effects), druglikeness and drug-score. Druglikeness was calculated based on equation summing up score values of the fragments present in the molecule under investigation. The fragments were identified from a list of 5300 distinct substructure fragments with associated druglikeness scores [25]. Drug-score was calculated combining the druglikeness, cLogP, logS, molecular weight and toxicity risks data [25].

### 3.5. Analgesic and Anti-Inflammatory Murine Models

#### 3.5.1. Animals

Experiments were conducted using Swiss mice obtained from the BIOCEN—UFAL breeding unit, weighing 20–30 g each, males, adult, with 6–8 weeks of age, distributed in groups up to 6–8 animals for treatment. Wistar rats (130–170 g), males, were used in the experiment of induction of arthritis. The animals were maintained with free access to food and water and kept at 25–28 °C under a controlled 12 h light/dark cycle. All animals were manipulated according to the norms established by the Ethics Commission—UFAL for handling animals (Protocol number: 14/2013).

#### 3.5.2. Acetic Acid-Induced Abdominal Constriction Test

The peripheral analgesic activity was evaluated in male mice using the acetic acid-induced writhing test [23]. The acetic acid-induced abdominal constriction test was carried out as described previously by Coolier, 1968 with minor modifications in groups of six animals. In order to induce pain in mouse peritoneal cavity, 0.6% of acetic acid (10 mL/Kg) was injected intraperitoneally, 40 min after the oral administration of 3-amino-thiophene-2-acylhydrazone derivatives **5a**–**i** (dose = 100, 30, 10, 3 and 1 µmol/Kg) and the standard dipyrone (dose = 100, 30, 10, 3 and 1 µmol/Kg). The ID_50_ was calculated by nonlinear regression. The abdominal constriction resulting from the injection of acetic acid consists of a contraction of the abdominal region together with a stretching of the hind limbs or all limbs. The number of abdominal constrictions was counted cumulatively over a period of 20 min, commencing 5 min after acetic acid administration. The animals were then placed immediately to individual in a transparent plastic box. Anti-nociception response was indicated by the reduction in the mean of 3-amino-thiophene-2-acylhydrazone derivatives **5a**–**i** number of abdominal constrictions in the test groups compared to the control group. Dipyrone was used as reference drugs while control group received vehicle (Arabic gum) that was used to dissolve compounds.

#### 3.5.3. Formalin Test

The method used for this test was similar to that described by Hunskaar and Hole [24] with minor modifications. Adult Swiss mice were divided in groups of six mice each and pretreated with 3-amino-thiophene-2-acylhydrazone derivatives **5a**–**i** (dose = 30 µmol/Kg, p.o.) or indomethacin (dose = 10 µmol/Kg, p.o.). Forty minutes after this treatment they were administered with 20 µL of a 2.5% solution of formalin, subcutaneously under the plantar surface of the left hind-paw. Using a chronometer, the total time spent in licking and biting the injected paw is recorded, quantifying thus the nociceptive behavior. Anti-nociceptive effect was determined in two phases, the early phase from 0 to 5 min and the late phase 15 to 30 min with a 10 min lag period in between both phases.

#### 3.5.4. Carrageenan-Induced Peritonitis

Peritoneal inflammation in male mice was produced according to the method described by Ferrándiz and Alcaraz, 1991 [25]. Carrageenan was freshly prepared (10 mg/mL) in sterile 0.9% w/v saline, and 250 μL were injected i.p., After 4 h, the animals were killed by cervical dislocation. The peritoneal cavity was washed with 3.0 mL cold PBS, and after a gentle manual massage, the exudate was retrieved and its volume was measured. The number of recruit leukocytes to the peritoneum was counted in a Newbauer chamber and results were expressed as cells × 10^6^/mL. The 3-amino-thiophene-2-acylhydrazone derivatives **5a**–**i** were tested orally in doses of 100, 30, 10, 3 and 1 µmol/Kg. The carrageenan group (Arabic gum, p.o.) and the reference drug (indomethacin, 100, 30, 10, 3 and 1 µmol/Kg, p.o.) were administered 30 min before the carrageenan injection. These five doses were perfomed in order to calculate the ID_50_. The ID_50_ was calculated by nonlinear regression. In the negative control group, animals have just received the same dose of a vehicle (arabic gum, p.o.) 30 min before the saline injection by intraperitoneal route.

#### 3.5.5. Arthritis Model Induced by Complete Freund’s Adjuvant (CFA) in Rats

Before the onset of arthritis, Wistar rats were randomly divided separately into five groups: the normal control group, the AA model group, the positive control group (*i.e.*, dexamethasone; 5 µmol/Kg, pathway orally, daily), **5a** (50 µmol/Kg, pathway orally, daily), **5d** (50 µmol/Kg, pathway orally, daily), and Complete Freund’s adjuvant (CFA) (0.2 mL/paw) was injected into the right paw of the rats. The CFA solution was prepared as a 1 mg/mL suspension of heat-killed *Mycobacterium butyricum* in 0.85 mL of paraffin oil and 0.15 mL of mannide monooleate (Sigma-Aldrich) [26]. At 14 days after the CFA injection the animals received during seven days treatment with derivatives **5a**, **5d** and dexamethasone. The paw volumes were measured on the 1st, 14th, 15th, 16th, 17th, 18th, 19th, 20th and 21st days using a digital caliper (Mitutoyo). Mean paw volumes were obtained daily for treated groups and compared with the paw volumes of the positive control group. At the 21st day the animals were anesthetized with a solution ketamine/diazepam (75 mg/Kg and 5 mg/Kg, i.p.), blood was collected by cardiac puncture for biochemical measurements using an Aspartate aminotransferase (AST), Alanine aminotransferase (ALT), Urea and Creatinin, methods kit (Doles-Brazil). After collection deeper anesthesia was applied to euthanize the animals. The arthritic paw was collected for histological analysis and the spleen was collected for weighing.

#### 3.5.6. Biochemical Measurements

The blood was collected by cardiac puncture using 50 µL of EDTA as an anticoagulant. The blood was centrifuged after collection was centrifuged at 1500 rpm for 5 min. After centrifugation the serum was aliquoted and used for carrying out the biochemical levels of urea, creatinine, AST and ALT. The level of AST/ALT was performed as follows. Two hundred µL of working reagent was placed in the wells of a 96 well plate, 20 µL of serum was added and homogenized performed as reading spectrophotometer wavelength at 340 nm. This was done with the initial reading and repeated three readings at intervals of 1, 2, and 3 min to calculate the Delta Abs. The formula used to calculate the dose of ALT/AST is below:

ALT UI/L = ΔA/minute × 1746

AST UI/L = ΔA/minute × 1746

The urea was carried expressed as follows: 100 µL of reagent 1 was placed in the cavity of a 96 well plate, and 5 µL of urease and 1 µL of serum sample or standard were added. The plate was incubated in a spectrophotometer for 5 min at 37 °C and then added to 100 µL of reagent 2. After that the reading plate with a wavelength of 600 nm was performed. The quantity of urea was calculated following the formula below:

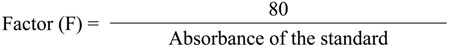

Urea (mg/dL) = Absorbance test × F

The serum creatinine test was performed according to the following protocol: 200 µL of working reagent was aliquoted into a 96-well plate, and 20 µL of serum were added. The plate was incubated for 30 s in the spectrophotometer. After incubation took place after the initial reading and the final reading of 60 s. The formula used to calculate the amount of creatinine in the samples is as follows:

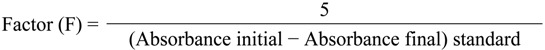

Creatinine (mg/dL) = (Abs ini − Abs fin) sample × F

#### 3.5.7. Macroscopic Analyses of the Stomach

After euthanasia, the stomachs were removed for macroscopic examination of the occurrence of lesions with daily treatment for 7 days with dexamethasone (5 µmol/Kg, pathaway orally), **5d** (50 µmol/Kg, pathway orally), and **5a** (50 µmol/Kg, pathway orally), The mucosal damage was examined by means of a magnifying glass. For each stomach, the mucosal damage was assessed according to the following scoring system [28].

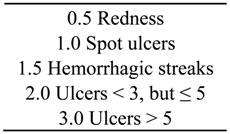



#### 3.5.8. Spleen Weight

In order to evaluate a possible immunosuppression promoted by the daily treatment for 7 days with dexamethasone (5 µmol/Kg, orally), **5d** (50 µmol/Kg, orally), and **5a** (50 µmol/Kg, orally), after euthanasia we proceeded with the removal of the spleen. The spleen was washed with PBS and weighed on an analytical balance. Treated groups were compared to the arthritic group [31,32].

#### 3.5.9. Statistical Analysis

Data obtained from animal experiments are represented by mean ± standard error of the mean (mean ± S.E.M.). Statistical differences between the treated and the control groups were evaluated by test t of Student or ANOVA in the tutorial Prisma^®^. Values were considered significant if * *p* < 0.05, ** *p* < 0.01 and *** *p*< 0.001.

## 4. Conclusions

In summary a series of functionalized 3-aminothiophene-2-acylhydrazone derivatives **5a**–**i** were designed and synthesized. These new *N*-acylhydrazone derivatives showed potent analgesic and anti-inflammatory activities, with **5a** and **5d** standing out in this respect. These compounds were active in acute and chronic inflammation models. After animals’ daily treatment for seven days with **5a** and **5d**, with a dose of 50 µmol/Kg by oral administration, they were unable did not present renal or hepatic toxicity. Moreover, **5d** didn’t demonstrate an immunosuppressive profile. Taken together, these data suggest the 3-aminothiophene-2-acylhydrazones **5a** and **5d** are new non-toxic, analgesic and anti-inflammatory lead-candidates. The mechanism of action of these two bioactive compounds is now being investigated.
